# The combined impact of cold smoking and natural antioxidants on quality and shelf life of dolphinfish (*Coryphaena hippurus*) fillets

**DOI:** 10.1002/fsn3.946

**Published:** 2019-03-18

**Authors:** Concetta Maria Messina, Gioacchino Bono, Rosaria Arena, Mariano Randazzo, Maria Morghese, Simona Manuguerra, Laura La Barbera, Fatih Ozogul, Saloua Sadok, Andrea Santulli

**Affiliations:** ^1^ Laboratorio di Biochimica Marina ed Ecotossicologia Dipartimento di Scienze della terra e del Mare DiSTeM Università degli Studi di Palermo Trapani Italy; ^2^ Istituto per l'Ambiente Marino Costiero Consiglio Nazionale delle Ricerche Mazara del Vallo Italy; ^3^ Istituto di Biologia marina Consorzio Universitario della Provincia di Trapani Trapani Italy; ^4^ Department of Seafood Processing Technology Faculty of Fisheries Cukurova University Adana Turkey; ^5^ Laboratoire de Biotechnologie Bleu et Bioproduits Aquatiques (B3Aqua) INSTM, Centre La Goulette Tunis Tunisie

**Keywords:** antioxidants, cold smoking, *Coryphaena hippurus*, dolphinfish, shelf life

## Abstract

This study aimed to produce new value‐added products from dolphinfish (*Coryphaena hippurus*) as fillets when using cold smoking combined with natural antioxidants, obtained from *Halocnemum strobilaceum* a plant belonging to halophytes. The fillet treatments were controlled (untreated), immersed in standard brine (STD), treated with NaCI and antioxidant before freezing (Aox‐pre) and treated with NaCl and antioxidant after thawing (Aox‐post). The quality of dolphinfish fillets was assessed using sensory, biochemical, microbiological, and technological parameters. Treating fillets with antioxidants before freezing and cold smoking (Aox‐pre) enhanced significantly the shelf life, as well as improved the biochemical, microbiological, and sensory aspects of the product. Aox‐pre fillets had the lowest peroxide values (PV) and malondialdehyde (MDA) content, demonstrating that the immediate treatment of fillets with the polyphenols protected lipids from peroxidation. The smoking process, together with antioxidant treatment, significantly reduced microbial counts after 15 days of storage, compared to the control. Overall, combining antioxidant treatments with cold smoking has a positive effect on the quality of Aox‐pre fillets with respect to both sensory components and biochemical parameters associated with shelf life. Consequently, Aox‐pre treatment enhances the marketability of this species, promoting local and seasonal products, contributing to costal fisheries sustainability.

## 1 INTRODUCTION

A variety of ecological and economic factors influence the global dynamics of fish species and coastal fisheries at the European level (Tacon & Metian, 2009; Witkin, Dissanayake, & McClenachan, 2015). Ecological factors include a decline of target species at higher trophic levels and increased availability of underutilized coastal fisheries species (UFS) from lower trophic levels. Economic factors include growing trends in imports and stagnation of the price of local products. In parallel, the seafood product market in Europe has grown significantly due to an increase in the average unit value of seafood products. This increase in the value‐added products has the potential to generate considerable demand for nonquota UFS, especially given the supply constraints of such quotas (Fagan, Ronan, & Michelle, 2006) and the dwindling stocks of traditional fisheries species. This shortfall has created the demand for high‐quality fillets, in addition to seafood products. Many UFS provide high‐quality fillets that are comparable to those obtained from commercial species (Gormley & Fagan, 2005). Therefore, it is necessary to explore the potential of UFS as value‐added products in the form of fillets or portions, as this opportunity could help diversity in European fisheries, which is one of the main goals of sustainable fisheries (Messina, Bono, Renda, La Barbera, & Santulli, 2015; Witkin et al., 2015).

The smoking process is useful for preserving the quality of seafood and, today, can be performed using both traditional and innovative techniques. Smoking delays microbial growth and oxidative processes through the synergic action of salt, smoke compounds, and dehydration (Fuentes, Fernández‐Segovia, Barat, & Serra, 2010). Smoke components, such as aldehydes, ketones, alcohols, acids, hydrocarbons, esters, phenols, and ethers, deposit on the surface of fillets and later penetrate into the muscle and are responsible for the final color and taste of products (Gómez‐Estaca, Gómez‐Guillén, Montero, Sopelana, & Guillén, 2011). Cold smoking is a mild, noninvasive, and useful method that improves the shelf life of products, without changing them radically, as it is usually carried out at temperatures that do not exceed 30°C; furthermore, it can provide a way of diversified, high value‐added products for certain species that are not available all over the year and its consumption as fresh product is limited (Gómez‐Guillén, Gómez‐Estaca, Giménez, & Montero, 2009).

It is well known that during storage, due to the high content of omega‐3 polyunsaturated fatty acids (PUFA), fish products are susceptible of peroxidation that can modify both sensorial characteristics and nutritional quality of fish (Maqsood, Benjakul, & Shahidi, 2013); for this reason, in the last years, some research on transformed fish highlighted the possibility to add natural antioxidants from plants, not only to extend shelf life but also to positively modify some sensory characteristics (Barbosa‐Pereira et al., 2013; Messina et al., 2015, 2016; Ozogul et al., 2017). In particular, it was demonstrated the effectiveness of some natural phenolic compounds in delay the spoilage processes in many fish species (Maqsood et al., 2013).

In this study, the combined effects of cold smoking and natural antioxidants on sensory, physical–chemical, nutritional, biochemical, and microbiological properties of dolphinfish (*Coryphaena hippurus*) fillets were compared according to previous approaches. (Gibbons, Mathew, & Gray, 1999; Messina et al., 2015; Miftakhova, Burasheva, Abilov, Ahmad, & Zahid, 2001).

The common dolphinfish, *C. hippurus*, is a highly valuable fish species in the Mediterranean countries, originated from coastal, artisanal fisheries (Messina et al., 2015; Potoschi, Cannizzaro, Milazzo, Scalisi, & Bono, 1999), that gives a good fillet yield and, usually, does not reach high prices. This species may be an alternative to other smoked species, such as salmon, mackerel, and trout (Gómez‐Estaca, Gómez‐Guillén, & Montero, 2007). Thus, smoking and other preservation techniques could be useful for expanding the availability of common dolphinfish for seafood processing sector.

## 2 MATERIALS AND METHODS

### 2.1 Fish sampling, processing, and pretreatment with antioxidants

A total of 24 specimens of *C. hippurus* (average length: 60.38 ± 4.21 cm; average weight: 1,161.88 ± 279.91 g) were collected in autumn by a coastal fishery boat's in Trapani (Italy) and were transferred in cold containers to the laboratory, within few minutes. The fish were then headed, gutted, cleaned, and filleted. Then, the fish were lightly wiped and stored under vacuum in Foodsaver (HDPE) and nylon bags (http://www.gopack.it), and subjected to rapid freezing at −35°C and maintained in that condition for 24 hr.

One lot of fillets (Aox‐pre, n = 12), before to be frozen, was treated for 2 min with a 1% aqueous solution of polyphenols obtained from the halophytes *Halocnemum strobilaceum*.

This plant is a local halophyte, that grows abundantly in areas close to the local salt plant, and has strong antioxidant properties due to its polyphenol content.

The antioxidant solution was prepared according to a previously described method (Messina et al., 2015). Briefly, 10 g of dried and powdered plant was extracted with 100 ml of distilled water for 24 hr. The sample was then filtered and lyophilized (Sung et al., 2009). The final solution of *H. strobilaceum* (HAL) was prepared by dissolving 10 g of lyophilized extract in 1,000 ml of distilled water, having a polyphenols content equal to 500 mg gallic acid equivalents (GAE)/L (Messina et al., 2015).

The next day, all samples were removed from the bags, placed in air‐permeable LDPE bags, and thawed at 4°C for 8 hr, until the smoking process.

### 2.2 Salting and smoking

**Figure 1:**
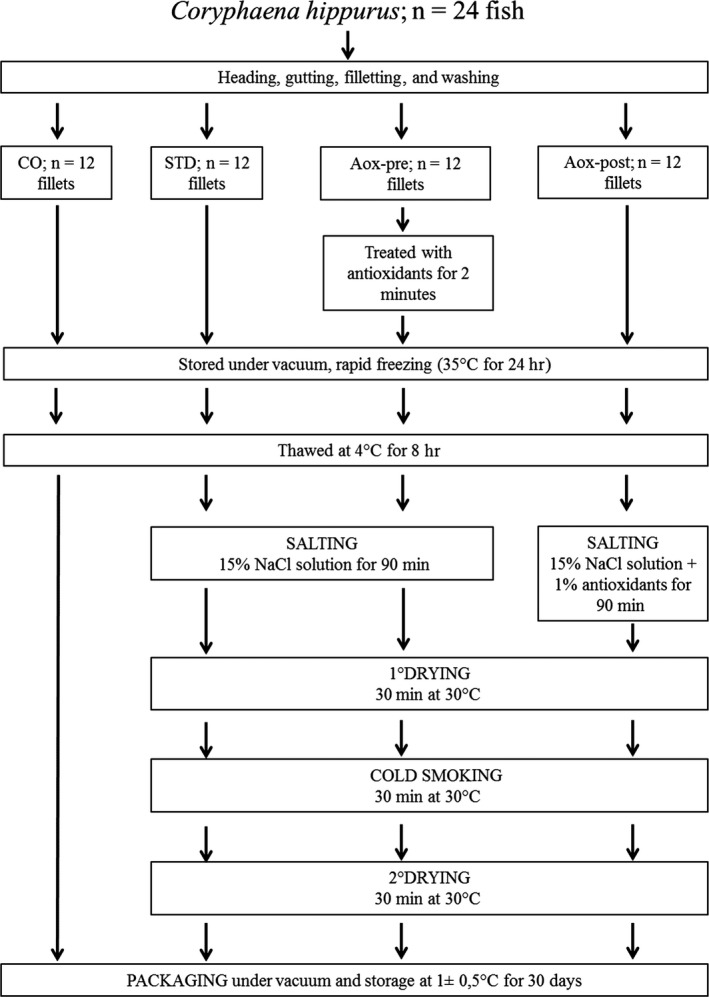
Flowchart of the experimental design

The process was separated into four steps: brine salting, first drying, smoking, and second drying (Figure 1). The thawed fillets were separated into three lots: the first lot was immersed in standard brine (STD), composed of 15% NaCl solution; the second lot was represented by fillets treated with antioxidants before freezing (Aox‐pre) and after treated with the same standard brine solution (15% NaCl); and the third lot was treated with the same standard brine with 1% antioxidants (Aox‐post). The remaining 12 fillets (control Co) were simply thawed and maintained at 4°C for all the trial in order to compare the shelf life parameters of the smoked product with the raw refrigerated product.

Salting was performed for 90 min (1/4, w/v fillets/brine ratio), and the fillets were then dried for 30 min at 30°C. Subsequently, cold smoking was performed using a Moduline oven model FA082E (Scubla srl, Remanzacco (Ud), Italy) for 30 min at 30°C, employing a mixture of the hacks pane (*Fagus* spp.) (Scubla srl, Remanzacco (Ud) Italy) and smoke shavings and Segamehl smoked flour (Scubla srl, Remanzacco (Ud) Italy). After the smoking process, all of the fillets were dried again under the previously described conditions.

### 2.3 Storage and sampling

At the end of the process, all the fillets were sealed in vacuum bags and stored at 1 ± 0.5°C for 30 days. Two fillets from each treatment group were analyzed after thawing (T0) at regular intervals (T1 = day 1; then, T6, T15, T21, T30) for each sampling day. The effects of the smoking process combined with natural antioxidants on quality of dolphinfish fillets were assessed by using a multidisciplinary approach involving sensory, physical–chemical, biochemical, and microbiological parameters. After the sensorial and instrumental analyses, the fillets were cold‐homogenized in order to be analyzed for the others parameters related to quality and shelf life.

### 2.4 Physical–chemical parameters

#### 2.4.1 Color

The analysis on the fillets was carried out using a Konica Minolta colorimeter (Osaka, Japan), and the results were reported according to the CIE system (CIE 1976). L*, a*, b*, and ΔE* representing lightness, redness/greenness, yellowness/blueness, and the total difference in color were reported.

ΔE* (CIE 1986) was calculated as:

$$\Delta E^{*}=\sqrt{{\left(\Delta L^{*}\right)}^{2}+{\left(\Delta a^{*}\right)}^{2}+{\left(\Delta b^{*}\right)}^{2}}$$

where ΔL*, Δa*, and Δb*are the differentials among the color parameters of the samples over the shelf life and the color parameters of the samples at T0.

#### 2.4.2 Texture

Two small fragments (1.8 cm Ø), obtained from the same portions of each fillet, were used. The analysis was performed at room temperature using an Instron Texture Analyzer Mod. 3342 (Turin, Italy).

The measured parameters were Hardness (N) and Young modulus or modulus of deformability (N/mm^2^) as the force and the slope of the curve at 50% compression, respectively (Orban, Sinesio, & Paoletti, 1997).

#### 2.4.3 Water holding capacity (WHC)

Water holding capacity was determined using the method described by Gómez‐Guillén, Montero, Hurtado, and Borderías (2000) with some modification. Briefly, chopped muscle (2 g) was placed in a centrifuge tube along with a paper filter and centrifugated at 500 g, for 10 min at 10°C. The aqueous plus fatty fraction (AF) (retained in the filter) was dried at 50°C until the constant weight. The fat release was calculated as: dry weight of AF × 100/initial weight of sample.

Water holding capacity (expressed as a percentage) was calculated as the difference between the percentage of the initial water content in the muscle and the water released after the centrifuge. The samples were analyzed in triplicate.

#### 2.4.4 Muscular pH

The muscular pH of the fillet was measured at three points along the lateral line with a Crison pH meter (Barcelona, Spain) equipped with a BlueLine pH 21 Schott Instruments (Weilheim, Germany) combined electrode.

### 2.5 Proximate composition and biochemical parameters related to the shelf life

The moisture content was assessed in the homogenate using the AOAC method (AOAC, 1990), and crude protein content was measured using the Kjeldahl method by multiplying the percentage of the nitrogen by the factor of 6.25 (AOAC, 1990). The total lipids (TL) were determined according to Folch, Lees, and Stanley (1957), and the fatty acid (FA) methyl esters were determined by the method of Lepage and Roy (1984); the gas chromatography was carried on under the conditions described in Messina, Renda, La Barbera, and Santulli (2013). For total polyphenol determination, the analyses were performed on samples from all lots after thawing or immediately after the smoking process. The total phenol contents were determined using the method described by Gómez‐Estaca, Gómez‐Guillén, and Montero (2014). The results were expressed as mg of phenol/g muscle. All analyses were performed in triplicate.

The peroxide values (PV) were calculated by the AOAC method (James, 1995), and the production of thiobarbituric acid reactive substances (TBARS) was determined using the method described by Botsoglou et al. (1994). The total volatile basic nitrogen (TVBN) was measured according to the EU Commission Decision 95/149/EC (EEC, 1995).

### 2.6 Microbiological analyses

Approximately 10 g of fillet was aseptically weighed into a sterile bag containing 90 ml sterile peptone water (PW) (0.1% w/v). The samples were homogenized for 6 min in a Stomacker Astori BR400 (Brescia, Italia). Then, a series of decimal dilutions were made and 0.1 ml of each dilution was plated on tryptone glucose yeast agar plates (tryptone 5 g/L, yeast extract 2.5 g/L, glucose 1 g/L, agar 9 g/L, pH 7 ± 0.2). Three plates were prepared for each dilution and were incubated at 37°C for 72 hr for mesophilic bacteria and at 6°C for 10 days for psychrophilic bacteria (Gökalp, 1986). Counts were made, and the results were given as Log (CFU/g).

### 2.7 Sensory analysis

The assessments were performed on fillets by a 12‐member panel according to the protocol developed by Gómez‐Guillén et al. (2009). Before the evaluation sessions, three scoring categories were defined: agreeable, neutral, and disagreeable. As a reference sample, a freshly smoked fillet that had been assessed as agreeable for all of the identified parameters (color, odor, flavor, firmness, juiciness, and acceptability) was used. The flavor evaluation was carried out up to the 6th day of storage. The results are presented as the percentage of panelists that assigned each of the three scores to each characteristic.

### 2.8 Statistical analysis

Statistical differences were evaluated for each parameter with analysis of variance (ANOVA). The differences among the mean values were assessed using the Student–Newman–Keuls test. The degree of heterogeneity was measured by the Cochran test (Underwood, 1997). The univariate analysis was done using STATISTICA (version 8.0, Statsoft Inc., USA).

## 3 RESULTS AND DISCUSSION

### 3.1 Physical–chemical parameters

Smoked dolphinfish has a characteristic taste and smoky flavor that might represent an innovative and high valued product for the local fishing industry. Smoked fish is subjected to different processing steps that alter the physical and chemical properties of the raw material, such as pH, WHC, color and texture and; for this reason, the regulation of the physicochemical parameters of the finished product is of great importance for sensory features and shelf life (Fuentes et al., 2010).

#### 3.1.1 Color

**Figure 2:**
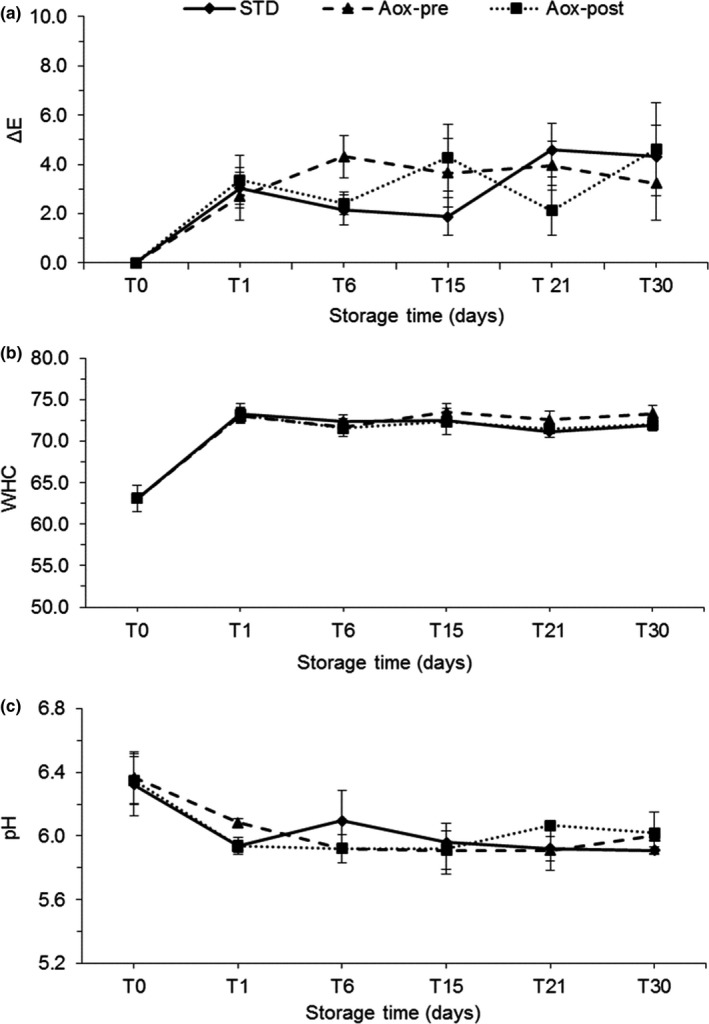
Physical–chemical parameters of cold‐smoked *C. hippurus* fillets during 30 days of storage; STD: immersed in standard brine; Aox‐pre: treated with NaCl solution + antioxidant before freezing; Aox‐post: treated with NaCl solution + antioxidants after thawing. (a): Δ*E*; (b): Water holding capacity (WHC); (c): pH

Color is the main parameter of quality that influences the consumers before purchase. The color measurements were made directly on part of the fillet that the consumer is able to view before purchase, using the packaging as the background, to obtain color measurements similar to those perceived by consumers before purchase (Fuentes et al., 2010). The smoking process causes significant variation to the color parameters. For instance, this study showed that L*, a*, and b* declined significantly (*p* < 0.05) in all treatments (data not shown). As indicators of the total differences in color, the Δ*E* value (Figure 2a) of the Aox‐pre fillets differed to that of the Aox‐post fillets with a uniform pattern in color variation during storage compared to STD group (immersed in standard brine).

#### 3.1.2 Texture

Smoking process also causes a considerable increase in all textural parameters, supporting that previous studies reported on other fish species (Birkeland, Rørå, Skåra, & Bjerkeng, 2004; Fuentes, Fernández‐Segovia, Serra, & Barat, 2012; Gómez‐Guillén et al., 2000; Regost, Jakobsen, & Rørå, 2004; Sigurgisladottir, Sigurdardottir, Torrissen, Vallet, & Hafsteinsson, 2000). Some of these changes, such as hardness, are due to water loss during the smoking process.

The results of the texture analysis (Young's Modulus and Hardness) are shown in Table 1. Young's Modulus values did not significantly differ among the treatments or during the storage period (Table 1). In comparison, the Hardness value significantly increased until the 15th day of storage (Table 1), but no significant differences were observed among treatments. This phenomenon occurred due to the combined effect of salting and drying causing the meat to harden (Fuentes et al., 2010). After 15 days of storage, the trend reversed due to the degradation processes, which was mainly related to autolytic phenomena and to the denaturation of proteins in the muscle tissue, resulting in a progressive reduction in the hardness of the tissue (Hernández, Martínez, & García García, 2001; Shouchun et al., 2010).

**Table 1:** Parameters related to texture—Young Modulus (N/mm^2^) and Hardness (N)—measured in cold‐smoked *C. hippurus* fillets during the 30 days of storage

		T0	T1	T6	T15	T21	T30
Young modulus (N/mm^2^)	STD	0.17 ± 0.07	0.23 ± 0.05	0.23 ± 0.06	0.29 ± 0.05	0.23 ± 0.07	0.24 ± 0.08
	Aox‐pre	0.17 ± 0.07	0.23 ± 0.07	0.24 ± 0.04	0.28 ± 0.09	0.20 ± 0.09	0.20 ± 0.05
	Aox‐post	0.17 ± 0.07^a^	0.18 ± 0.08^a^	0.27 ± 0.07^ab^	0.32 ± 0.07^b^	0.25 ± 0.06^ab^	0.23 ± 0.05^ab^
Hardness (N)	STD	5.19 ± 1.95^a^	8.43 ± 1.97^ab^	9.96 ± 2.24^ab^	13.00 ± 2.05^b^	8.39 ± 3.85^ab^	9.65 ± 1.96^ab^
	Aox‐pre	5.19 ± 1.95^a^	6.81 ± 3.00^a^	9.19 ± 3.82^ab^	13.62 ± 6.21^b^	7.34 ± 3.76^ab^	8.30 ± 2.91^ab^
	Aox‐post	5.19 ± 1.95^a^	6.68 ± 2.93^a^	9.94 ± 2.46^ab^	12.98 ± 3.35^b^	8.98 ± 2.58^ab^	11.12 ± 3.28^ab^

Note

Data within a row that have different superscript letters are statistically different (*p* < 0.05). Each value represents the mean of three independent determinations.

Aox‐post: treated with NaCl solution + antioxidant after thawing; Aox‐pre: treated with NaCl solution + antioxidants before freezing; STD: immersed in standard brine.

#### 3.1.3 Water holding capacity

Water holding capacity is the ability of muscle to retain water, which is an important parameter from both industrial and consumer perspectives. WHC is closely related to changes that occur in muscle proteins and is considered a measure of the tenderness and juiciness of fish meat (Huss, 1995). In the current study, all treatments had similar values for WHC during chilled storage, which is an agreement with previous similar studies (Gómez‐Guillén et al., 2009). This parameter generally decreases with postmortem structural changes in the muscle (Kaale, Eikevik, Rustad, & Nordtvedt, 2014). However, in this study, the WHC values, showed in Figure 2b, remained stable throughout storage, based on the significant increase in Hardness. WHC values did not significantly differ among treatments or during the storage period, with an average value of 71.91 ± 1.21% at the end of storage.

#### 3.1.4 Muscular pH

Figure 2c shows the pH values of the different treatments. After 30 days of storage, the pH of cold‐smoked dolphinfish was lower (5.9 and 6.2) than that of untreated fillets (6.34; *p* < 0.05). Leroi and Joffraud (2000) found that salt and smoking caused the pH of salmon to decline, probably due to an increase in ionic force caused by the combined effect of salt and smoke. When comparing the trend of pH with WHC in this study (Figure 2b,c), both parameters appeared relatively unchanged with storage time. This finding was supported by Huss (1995), who stated that the changes of pH cause the partial denaturation of muscle proteins (especially when declining toward acid values), and the subsequent loss of water holding capacity.

### 3.2 Proximate composition and biochemical parameters related to the shelf life

The proximate composition and the polyphenol content of fresh and smoked dolphinfish fillets are shown in Table 2. The STD, Aox‐pre, and Aox‐post treatments presented a considerable amount of deposited phenols. This phenomenon arose because the smoke components, such as phenols, are deposited on the surface and later penetrate into the muscle (Gomez‐Guillen & Montero, 2007). Quantitative differences in the polyphenol content were found between three lots compared to the control, with the highest content being detected for Aox‐pre (*p* < 0.05). The nutritional values remain unchanged following smoking, except for content of ashes and water, which varied due to water loss. In fact, the preliminary salting and smoking process led to a reduction in moisture and an increase in ash and mineral contents, as observed in previous studies (Fuentes et al., 2012).

**Table 2:** Proximate composition (g/100 g wet weight) and polyphenol content (mg GAE/g) of *C. hippurus* fillets

	CO	STD	Aox‐pre	Aox‐post
Lipid	2.15 ± 0.47	2.18 ± 0.65	1.99 ± 0.20	2.01 ± 0.08
Moisture	76.98 ± 1.03	73.46 ± 0.59	74.92 ± 0.39	73.35 ± 0.58
Protein	18.55 ± 0.72	18.27 ± 0.86	18.37 ± 0.42	18.47 ± 1.07
Ash	1.31 ± 0.08	4.97 ± 0.82	4.00 ± 0.30	4.49 ± 0.37
mg GAE/g muscle	0.22 ± 0.02^a^	0.28 ± 0.06^a^	0.52 ± 0.05^b^	0.28 ± 0.05^b^

Note

Data within a row that have different superscript letters are statistically different (*p* < 0.05). Each value represents the mean of three independent determinations.

Aox‐post: treated with NaCl solution + antioxidant after thawing; Aox‐pre: treated with NaCl solution + antioxidants before freezing; CO: untreated group; STD: immersed in standard brine.

The total lipid content of the thawed fillets was 2.5 (g/100 g wet weight) (Table 2). The low total lipid content confirmed that dolphinfish is a low‐fat fish species (Messina et al., 2015). In general, the lower the fat content of a fish, the lower the susceptibility to peroxidation (Kostaki, Giatrakou, Savvaidis, & Kontominas, 2009; Tang, Kerry, Sheehan, Buckley, & Morrissey, 2001). FA composition of common dolphinfish fillets showed no significant difference among treatments at any stage of the experiment. This finding confirms that both natural antioxidant treatment and the cold smoking process do not alter the biochemical properties of dolphinfish.

The mean value of the data recorded from day 0 to day 21 for each treatment is showed in Table 3. All lots were characterized by high levels of n‐3 polyunsaturated fatty acids (PUFAs), followed by saturated FAs, monounsaturated FAs, and omega‐6 (n‐6) PUFAs (Table 3).

**Table 3:** Fatty acids classes (g/100 g) in *C. hippurus* fillets

	CO	STD	Aox‐pre	Aox‐post
Saturated	30.76 ± 0.96	31.07 ± 1.07	30.39 ± 0.81	30.20 ± 1.64
Monounsaturated	14.08 ± 1.03	13.66 ± 1.42	14.31 ± 0.43	13.22 ± 0.62
Tot n‐3	50.25 ± 1.66	51.91 ± 2.15	51.27 ± 0.96	52.49 ± 2.06
Tot n‐6	2.42 ± 0.20	2.10 ± 0.99	2.63 ± 0.22	2.86 ± 0.15
DHA	43.31 ± 1.82	45.16 ± 2.22	45.38 ± 1.84	43.88 ± 1.00
DHA/EPA	8.58 ± 0.60	9.52 ± 0.53	8.98 ± 0.42	9.24 ± 0.34

Note

Each value represents the mean of three independent determinations.

Aox‐post: treated with NaCl solution + antioxidant after thawing; Aox‐pre: treated with NaCl solution + antioxidants before freezing; CO: untreated group; STD: immersed in standard brine.

The high level of docosahexaenoic acid (DHA) (Co 43.31 g/100 g; STD 45.16 g/100 g; Aox‐pre 43.88 g/100 g; and Aox‐post 45.38 g/100 g) compared to other FAs confirmed the nutritional value of this species (Messina et al., 2015). The consumption of fish rich in n‐3 PUFAs, especially DHA and eicosapentaenoic acid (EPA), is associated with beneficial effects on cardiovascular health and visual function, in addition to lowering the occurrence of Alzheimer's disease (Arab‐Tehrany et al., 2012; Messina et al., 2015). Antioxidant treatment of different species fillets prior to freezing has been shown to prevent lipid oxidation (Hamre, Lie, & Sandnes, 2003).

**Figure 3:**
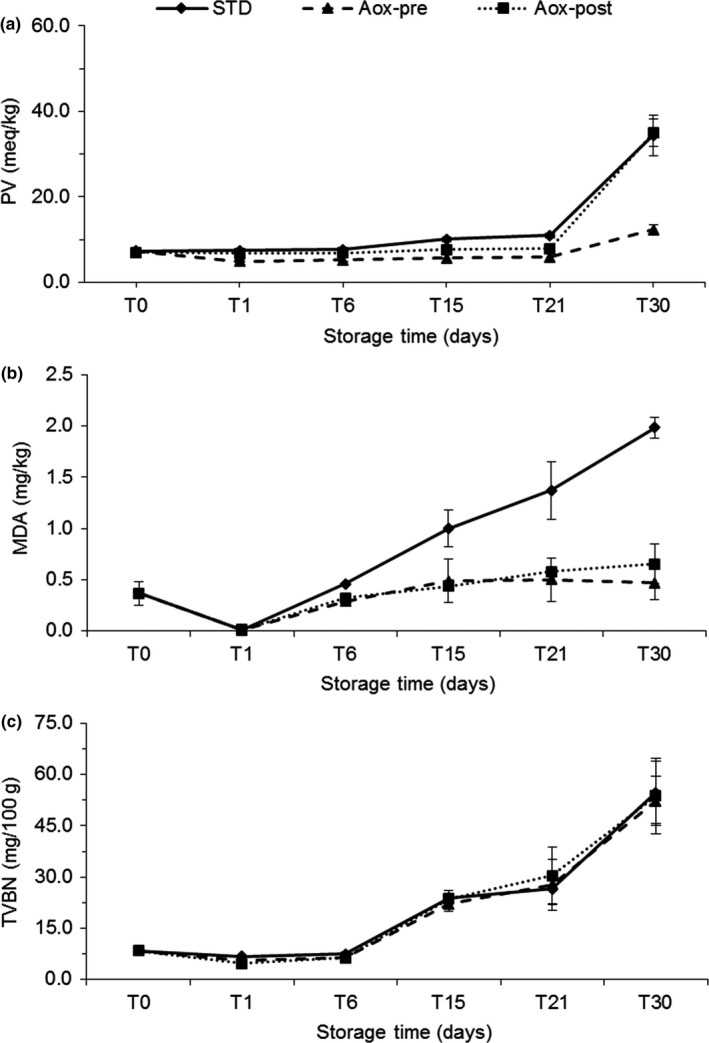
Biochemical parameters associated to the shelf life of cold‐smoked *C. hippurus* fillets during 30 days of storage: STD: immersed in standard brine; Aox‐pre: treated with NaCl solution + antioxidants before freezing; Aox‐post: treated with NaCl solution + antioxidant after thawing. (a): peroxide values (mEq/kg); (b): TBARS (mg MDA/kg); (c): TVBN (mg/100 g). Each point represents the mean of three independent determinations

Data on PV content are presented in Figure 3a. All of the values found at the end of the storage were <100 meq/kg kg (Figure 3a), which is considered the limit for human consumption (Gotoh & Wada, 2006; Gotoh et al., 2006). Thus, regardless of using the antioxidant extract of a plant, salt‐smoking techniques combined with vacuum packaging (i.e., without oxygen) significantly decreased the primary lipid oxidation and, consequently, enhanced the quality and shelf life of dolphinfish fillets. More specifically, during the first 21 days of storage, Aox‐pre had the best performance (max PV: 5.89 ± 1.25 meq/kg on day 21), followed by Aox‐post (max PV: 7.84 ± 0.74 meq/kg on day 21), and STD (max PV: 10.95 ± 0.52 meq/kg on day 21). Furthermore, at the end of storage, Aox‐pre batch fillets had the lowest values (max: 12.34 ± 1.21 meq/kg), demonstrating the preservative effect of the plant extract used before freezing.

This result was also supported by the good residual value of phenol content of Aox‐pre, which was significantly higher (0.52 ± 0.05 mg GAE/g muscle; Table 2) compared to Aox‐post, Co, and STD. These results confirm the beneficial roles of phenolic compounds in preventing lipid oxidation in smoked fish (Gómez‐Estaca et al., 2007; Gomez‐Guillen & Montero, 2007) as observed by the reported levels of TBARS (Figure 3b) in both treatments with antioxidants, during the storage process (Figure 3b). Malondialdehyde (MDA) is representative of the secondary oxidation phase and was present in low levels in the antioxidant‐treated fillets (Aox‐pre and Aox‐post) compared to STD, being also responsible of the sensory evaluation scores in all groups, confirming the influence of lipid peroxidation in altering the sensory properties of fish products.

Total volatile basic nitrogen values during storage are reported in Figure 3c. Evidence of deterioration was detected after 21 days of storage (Figure 3c). European legislation (European, 1995) has established an upper limit for TVBN, ranging from 25 to 35 mg TVBN per 100 g of muscle, depending on the species. Moreover, limits for processed fish do not exist at present and the upper limit used is equal to 35 mg for smoked dolphinfish fillets (Gómez‐Guillén et al., 2009). Assuming the same limit, these results showed signs of deterioration after 21 days of storage. After 30 days, none of the samples were suitable for consumption, with TVBN values ranging from 40 to 55 mg/100 g.

### 3.3 Microbiological analyses

**Figure 4:**
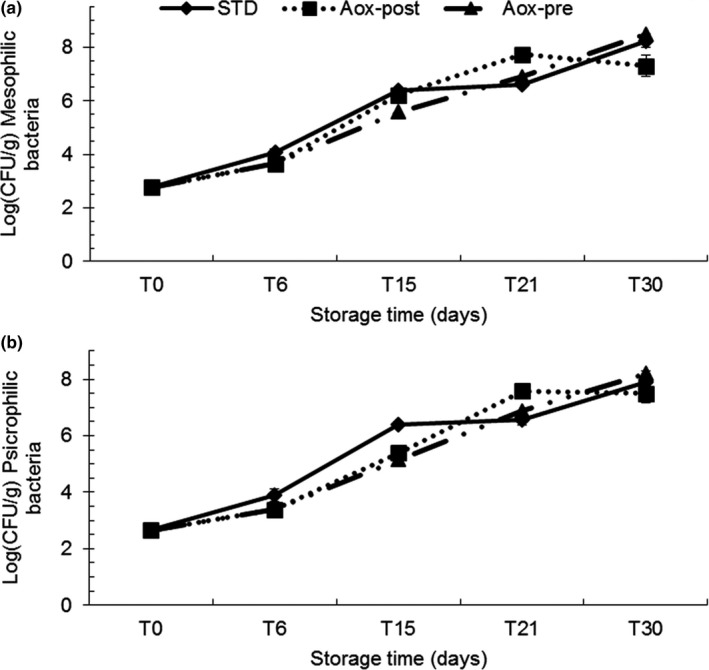
Microbiological parameters associated to shelf life of cold‐smoked *C. hippurus* fillets during 30 days of storage: STD: immersed in a standard brine; Aox‐pre: treated with NaCl solution + antioxidants before freezing; Aox‐post: treated with NaCl solution + antioxidant after thawing. (a): mesophilic (37°C); (b): psychrophilic (6°C). Each point represents the mean of three independent determinations

The total bacterial count confirmed the bacteriostatic effect of the cold smoking process. This phenomenon arises due to the synergistic action of the salt that is absorbed during the brine process and due to the deposition of polyphenols during smoking (Gómez‐Guillén et al., 2009; Oueslati et al., 2012). At T0, the initial bacterial load was found to be 2.764 ± 0.282 log (CFU/g) for mesophilic bacteria and 2.653 ± 0.184 log (CFU/g) for psychrophilic bacteria (Figure 4). During the first 15 days of storage at 4°C, no mesophilic or psychrophilic bacteria were detected. After 15 days of storage, STD had a higher bacterial charge than the antioxidant‐treated fillets (Aox‐pre and Aox‐post). However, up to 21 days of storage, all treatments had acceptable values, according to the current standard (<10^7^ CFU/g) (AFSSA, 2008; Decreto Ministeriale del 18/09/2002, 2002; FCD, 2009; Healt Protection Agency, 2009).

### 3.4 Sensory analysis

Sensory analysis is considered one of the best tool to determine the fish quality in the industry and consumer market (Loutfi, Coradeschi, Mani, Shankar, & Rayappan, 2015; Warm, Martens, Nielsen, & Martens, 2001). Sensorial aspects of quality in fish are closely related to the chemical and biochemical composition of the fish and to their variations during storage (Antoine et al., 2002; Du, Huang, Kim, Marshall, & Wei, 2000; Gómez‐Guillén et al., 2009; Messina et al., 2016). The temperature of preservation and the methods of processing contribute to preventing spoilage, preserving quality, and extending the shelf life of fish, as well as improving the overall quality (Masniyom, 2011; Mastromatteo, Conte, & Del Nobile, 2010; Messina et al., 2016). Thus, sensorial analysis is probably the best way to quickly estimate the shelf life of fish products (Leroi & Joffraud, 2000; Martinez, Salmerón, Guillén, & Casas, 2007).

**Figure 5:**
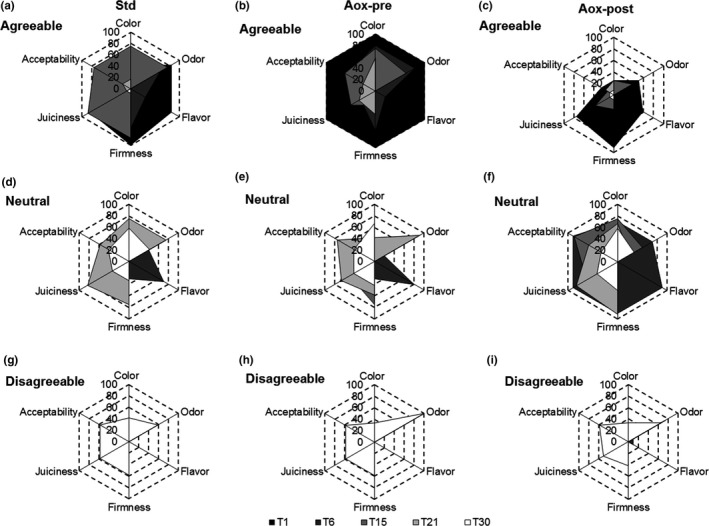
Sensory analysis of cold‐smoked *C. hippurus* fillets during 30 days of storage. The results of each attribute are expressed as the percentage of the panelists that evaluated them as agreeable, neutral, and disagreeable. (a): STD agreeable; (b): Aox‐pre agreeable; (c): Aox‐post agreeable; (d): STD neutral; (e): Aox‐pre neutral; (f): Aox‐post neutral; (g): STD disagreeable; (h): Aox‐pre disagreeable; (i): Aox‐post disagreeable. Each point represents the mean of three independent determinations

In our study, the methods of processing and the use of natural antioxidants effectively preserved the quality and shelf life of dolphinfish. The natural antioxidants used before freezing (Aox‐pre) had a noticeable effect at T1 (Figure 5b). All the panelists judged agreeable each parameter of the pretreated fillets (Figure 4b). At T6, the 80% of the panelists judged as agreeable the odor and color attributes of the Aox‐pre samples (Figure 5b). At T15, the highest percentage was obtained for the STD samples (Figure 5a) (from 75% to 85% for all attributes). At T21, over 50% of panelists (Figure 5d,e,f) assigned neutral scores for all attributes, with the exception of flavor, which was not monitored after T15. Thus, the results show that the use of natural antioxidants used before freezing (Aox‐pre) has a positive effect on the shelf life of smoked product. Similar results are being reported in other recent studies (Emir Çoban & Özpolat, 2013). Pretreatment with antioxidants had a significant positive effect on the entire shelf life period, improving also the sensory aspect of the product.

Sensory data give an indication of not only bacterial spoilage but also spoilage from chemical reactions, in particular lipid oxidation (Kristinsson, Danyali, & Ua‐Angkoon, 2007). According to Kristinsson et al. (2007), in our study, the combined treatment with cold smoking and antioxidant allows to maintain a lower microbial count and significantly lower values of PV and MDA (Figure 3a,b) determining different sensorial aspects of the products.

## 4 CONCLUSIONS

The combined treatment with cold smoking and antioxidant determined an overall amelioration of the quality traits and shelf life of dolphinfish fillets; the antioxidant treatment prevented the lipid peroxidation and this aspect influenced also the sensorial aspects of the products, compared to the untreated fillets. Accordingly, treatment of Aox‐pre improves the shelf life and the marketability of this species, promoting local and seasonal products and contributing to coastal fisheries sustainability.

## CONFLICT OF INTEREST

All authors declare no conflict of interest.

## ETHICAL STATEMENT

The work was conformed to Directive 2010/63/EU.
